# The Epidermal Growth Factor-like Domain of CD93 Is a Potent Angiogenic Factor

**DOI:** 10.1371/journal.pone.0051647

**Published:** 2012-12-18

**Authors:** Yuan-Chung Kao, Shinn-Jong Jiang, Wen-An Pan, Kuan-Chieh Wang, Po-Ku Chen, Hsi-Ju Wei, Wei-Sheng Chen, Bi-Ing Chang, Guey-Yueh Shi, Hua-Lin Wu

**Affiliations:** 1 Department of Biochemistry and Molecular Biology, College of Medicine, National Cheng Kung University, Tainan, Taiwan; 2 Cardiovascular Research Center, National Cheng Kung University, Tainan, Taiwan; 3 Center for Bioscience and Biotechnology, National Cheng Kung University, Tainan, Taiwan; Massachusetts General Hospital/Harvard Medical School, United States of America

## Abstract

Human CD93, an epidermal growth factor (EGF)-like domain containing transmembrane protein, is predominantly expressed in the vascular endothelium. Studies have shown that AA4, the homolog of CD93 in mice, may mediate cell migration and angiogenesis in endothelial cells. Soluble CD93 has been detected in the plasma of healthy individuals. However, the role of soluble CD93 in the endothelium remains unclear. Recombinant soluble CD93 proteins with EGF-like domains (rCD93D123, with domains 1, 2, and 3; and rCD93D23, with domains 2 and 3) were generated to determine their functions in angiogenesis. We found that rCD93D23 was more potent than rCD93D123 in stimulating the proliferation and migration of human umbilical vein endothelial cells (HUVECs). Production of matrix-metalloproteinase 2 increased after the HUVECs were treated with rCD93D23. Further, in a tube formation assay, rCD93D23 induced cell differentiation of HUVECs through phosphoinositide 3-kinase/Akt/endothelial nitric oxide synthase and extracellular signal-regulated kinases-1/2 signaling. Moreover, rCD93D23 promoted blood vessel formation in a Matrigel-plug assay and an oxygen-induced retinopathy model *in vivo*. Our findings suggest that the soluble EGF-like domain containing CD93 protein is a novel angiogenic factor acting on the endothelium.

## Introduction

Angiogenesis, which involves the formation of new blood vessels from pre-existing vessels, is a complex process that plays an important role in various physiological and pathological conditions, including embryonic development [1], wound healing [2], and tumor growth [3]. During angiogenesis, angiogenic factors activate and bind to the receptors on blood vessels. Subsequently, the activated endothelial cells secrete matrix-metalloproteinases (MMPs) to degrade extra-cellular matrix to allow pre-existing vessels to proliferate and sprout to neighboring vessels. The sprouting neo-vessels weave a connecting vessel web by tube formation [1]. In addition, activations of focal adhesion kinase (FAK), extracellular signal-regulated kinases-1/2 (ERK1/2), and phosphoinositide 3-kinase (PI3K)/Akt/endothelial nitric oxide synthase (eNOS) axes are responsible for the cell migration, proliferation, permeability, and homeostasis of the endothelium [4].

CD93 is composed of 652 amino acids and belongs to type-I transmembrane glycoprotein [5]. The structure of human CD93 is consisted of five distinct domains, including a unique C-type lectin-like domain (CTLD) (designated as D1), a tandem array of five epidermal growth factor (EGF)-like repeats (designated as D2), a Ser/Thr rich mucin-like domain (designated as D3), a 25-amino acid transmembrane domain (designated as D4), and a 47-amino acid cytoplasmic domain (designated as D5) [5]. Although the predicted molecular mass of CD93 is 68 kDa, its relative migration in SDS-PAGE under reducing conditions is 126 kDa, suggesting that it is heavily glycosylated [6]. Consistent with this notion, further study has shown that the cell surface expression of CD93 is stabilized by *O*-glycosylation [7]. CD93 is selectively expressed on myeloid cells (granulocytes and monocytes), platelets, stem cells, and mainly in endothelial cells [8], [9], [10], [11], [12]. Subsequent studies have demonstrated that in both humans and mice, CD93 is not present on tissue macrophages and that the predominant site of expression is the vascular endothelium [9], [13], [14]. In addition, it has been reported that during embryogenesis, mRNA and protein expression of mouse CD93 (also known as AA4) are first detected in the endocardium and the vascular endothelium in day 9 embryos. At later stages of development, the expression pattern of mouse CD93 is maintained by the formation of the capillary network invading various organ rudiments, suggesting that CD93 is involved in angiogenesis but not vasculogenesis [13].

Several soluble and cell surface-bound mediators including growth factors, cytokines, chemokines, proteolytic matrix-degrading enzymes, and cell adhesion molecules have been documented as angiogenic factors, which may promote neovascularization [15], [16]. Most of these angiogenic factors contain the EGF-like domain, which may display mitogenic effect on endothelial cells [16]. Previous reports have shown that the soluble CD93 fragments containing the EGF-like domain is released during inflammation in a peritonitis mouse model [17]. Moreover, elevated CD93 expression is also detected in the plasma of the patients who have overcome from coronary artery disease [18]. Theses clinical observations imply a clue to further investigate the impact of soluble CD93 on angiogenesis. However, whether soluble CD93 functions as an angiogenic factor and which domain is essential in angiogenesis are never addressed. In this study, we generated recombinant CD93 (rCD93) domain proteins, including domain 1 (rCD93D1), domains 2 and 3 (rCD93D23), and domains 1, 2, and 3 (rCD93D123), to examine the effect of these recombinant proteins on angiogenesis. The present study shows that the EGF-like domain of CD93 induces endothelial cell proliferation, migration, and signaling pathways *in vitro*, and stimulates angiogenesis *in vivo*.

## Results

### The Expression and Purification of rCD93 Domain Proteins

**Figure 1:**
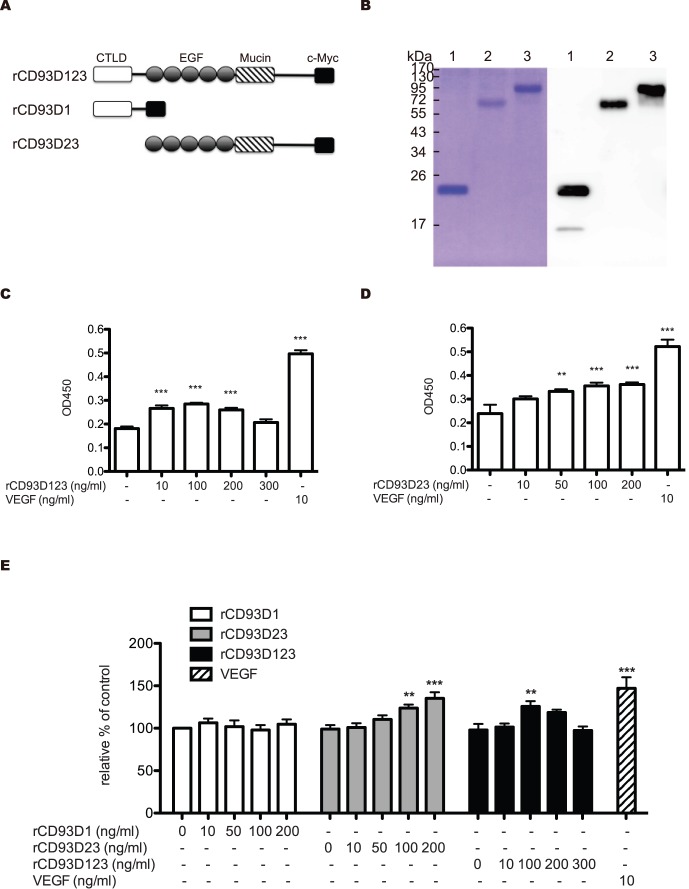
Effect of rCD93 domain proteins on HUVECs proliferation. (A) Illustration of rCD93D1, rCD93D23, and rCD93D123. CTLD, C-type lectin-like domain. EGF, EGF-like domain. Mucin, mucin domain. c-Myc, used as a tag. (B) Purified rCD93D1, rCD93D23, and rCD93D123 were subjected to SDS-PAGE (10%) under reducing conditions. Coomassie blue-stained purified rCD93D1 (lane 1), rCD93D23 (lane 2), and rCD93D123 (lane 3). Western blotting of purified rCD93D1, rCD93D23, and rCD93D123 with monoclonal mouse anti c-Myc Ab. HUVECs were incubated with different concentrations of (C) rCD93D123 or (D) CD93D23, or 10 ng/ml of VEGF for 48 h. Subsequently, the proliferation of HUVECs was measured by WST-1 reagent at OD450. (E) The cell numbers were counted. Each value represents the mean ± SD (n = 3), and similar results were obtained in three independent experiments. **, *p*<0.01; ***, *p*<0.001 vs. medium alone.

The rCD93D1, rCD93D23, and rCD93D123 (Fig. 1A) were prepared by a mammalian protein expression system. The purified rCD93D1, rCD93D23, and rCD93D123 were subjected to SDS-PAGE and assessed by western blotting with a monoclonal anti-c-Myc antibody (Ab). The theoretical molecular masses of rCD93D1 (20,346.76 Da), rCD93D23 (36,739.07 Da), and rCD93D123 (61,876.55 Da) were analyzed by the integrated bioinformatics database ExPASy, respectively. However, the molecular masses of rCD93D1, rCD93D23, and rCD931D23, as shown on SDS-PAGE (Fig. 1B), were 20, 71, and 95 kDa, respectively, which suggested that they were heavily glycosylated, as they were in their native state. The purified proteins were further identified by a liquid chromatography mass spectrometry/mass spectrometry (LC-MS/MS) (Fig. S1A).

### Effect of rCD93 Domain Proteins on Human Umbilical Vein Endothelial Cells (HUVECs) Proliferation

Effect of rCD93D23 and rCD93D123 on the HUVECs proliferation was evaluated in the presence of 1% fetal bovine serum (FBS) by a WST-1 assay and a cell counting manner. Both rCD93D23 and rCD93D123 stimulated HUVEC proliferation (Fig. 1, C–E), while rCD93D1 showed a nonsignificant mitogenic effect, suggesting that rCD93 containing an EGF-like domain possessed mitogenic activity.

### Effect of rCD93 Domain Proteins on HUVEC Migration

**Figure 2:**
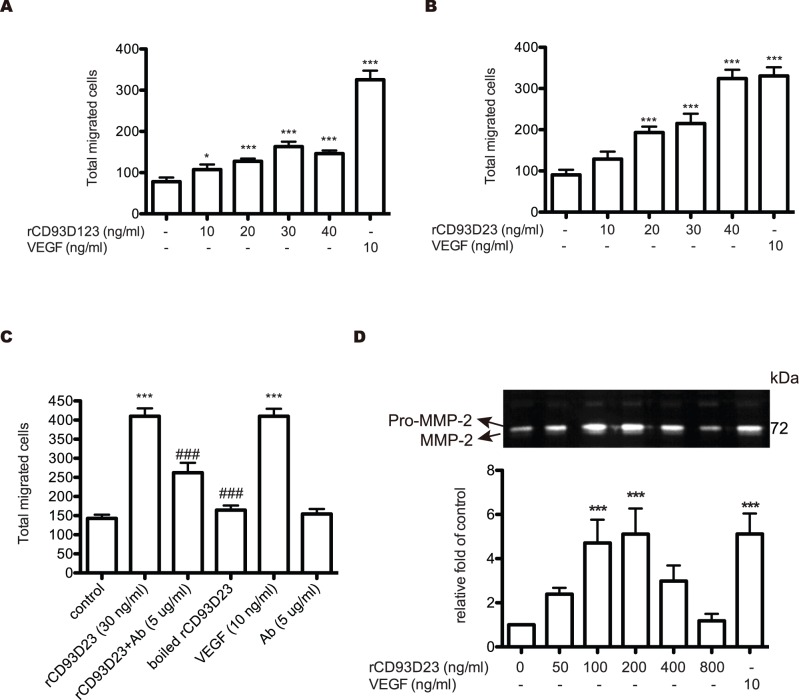
Effect of rCD93 domain proteins on the chemotactic migration and MMP secretion of HUVECs. A migration assay was performed in Transwell. (A) HUVECs (1 × 10^5^ cells) suspended in M199 supplemented with 1% FBS were added to the upper wells. The lower wells were filled with different concentrations of rCD93D123 (A) or rCD93D23 (B), or with 10 ng/ml of VEGF. (C) The lower wells were filled with rCD93D23 (30 ng/ml) in the absence or presence of CD93D23 Ab (5 µg/ml), boiled rCD93D23, or 10 ng/ml of VEGF. Each value represents the mean ± SD (n = 3), and similar results were obtained in three independent experiments. *, *p*<0.05; **, *p*<0.01; ***, *p*<0.001 vs. medium alone. ###, *p*<0.001 vs. rCD93D23. (D) The culture media of HUVECs treated with different concentrations of rCD93D23 or VEGF (10 ng/ml) were used in gelatin zymography. The similar results were obtained in three independent experiments. ***, *p*<0.001 vs. medium alone.

The chemotactic motility of HUVECs was assayed using Transwell to investigate the effect of rCD93D23 and rCD93D123 on HUVEC migration. rCD93D23 and rCD93D123 induced HUVEC migration in a dose-dependent manner, which reached maximum stimulation at a concentration of 30 ng/ml (Fig. 2, A and B). rCD93D1, however, had no effect on cell migration (data not shown). Since rCD93D23 had obviously higher mitogenic ability than rCD93D123 to stimulate cell proliferation (Fig. 1, C, D, E) and migration (Fig. 2, A and B), it was specifically applied to further experiments. When rCD93D23 was pre-mixed with rCD93D23 Ab at a concentration of 5 µg/ml or was boiled for 30 min (Fig. 2C), the chemotactic response induced by rCD93D23 was attenuated to a level comparable to that of the control. Moreover, rCD93D23 could induce expression and activation of MMP-2 in a dose-dependent manner as shown by a gelatin zymography assay (Fig. 2D). The results indicated that HUVEC migration was specifically induced by rCD93D23.

### Signaling Cascade Mediated by rCD93D23 in HUVECs

**Figure 3:**
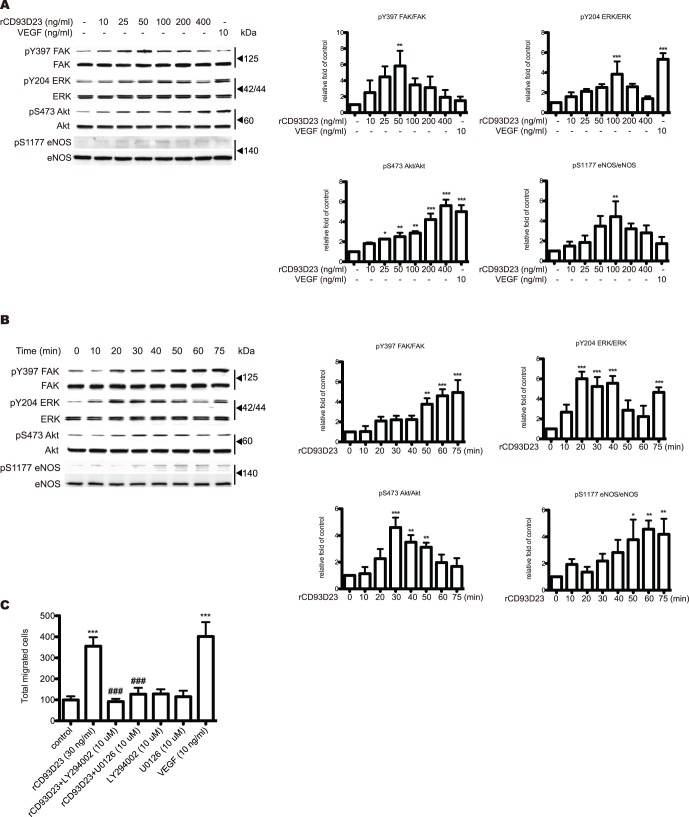
rCD93D23 induces HUVEC migration through the activation of Akt and ERK. (A) rCD93D23 dose-dependently induced FAK, ERK1/2, Akt, and eNOS phosphorylation. HUVECs were treated with different concentrations of rCD93D23 or VEGF (10 ng/ml) for 30 min. (B) rCD93D23 time-dependently induced FAK, ERK1/2, Akt, and eNOS phosphorylation in HUVECs. HUVECs were stimulated with 50 ng/ml of rCD93D23 for various time periods as indicated. The similar results were obtained in three independent experiments. *, *p*<0.05; **, *p*<0.01; ***, *p*<0.001 vs. control (C) HUVEC suspensions were pretreated with LY294002 (10 µM) or U0126 (10 µM). The lower wells were filled with rCD93D23 (30 ng/ml) or VEGF (10 ng/ml). Each value represents the mean ± SD (n = 3), and similar results were obtained in three different experiments. ***, *p*<0.001 vs. control. ###, *p*<0.001 vs. rCD93D23.

Since FAK, ERK1/2, and PI3K/Akt/eNOS pathways mediate endothelial cell proliferation and migration during angiogenesis, we next studied whether these signaling pathways were involved in rCD93D23-stimulated HUVECs by analyzing the levels of phospho- and total protein of the cell lysates with western blotting. rCD93D23 could stimulate FAK, ERK1/2, Akt, and eNOS phosphorylation in a dose-dependent manner (Fig. 3A). Furthermore, rCD93D23 at 50 ng/ml was used to assess the time-dependent activation of signaling cascades in rCD93D23-stimulated HUVECs. The degree of signal activation was altered with the time of treatment (Fig. 3B). These data indicated that rCD93D23, in addition to activating ERK1/2, also stimulated the PI3K/Akt/eNOS pathway in HUVECs. To further examine whether rCD93D23-stimulated HUVECs migration was through the ERK1/2 and PI3K/Akt/eNOS pathways, cells were pretreated with either 10 µM U0126 or 10 µM LY294002 for one hour prior to being stimulated with rCD93D23. The stimulatory effect of rCD93D23 on migration was blocked by U0126 or LY294002 (Fig. 3C). This result suggested that rCD93D23 mediated HUVECs migration was predominantly through the FAK, ERK1/2, and PI3K/Akt/eNOS pathways.

### rCD93D23 Induces Vascular Tube Formation *in vitro*

**Figure 4:**
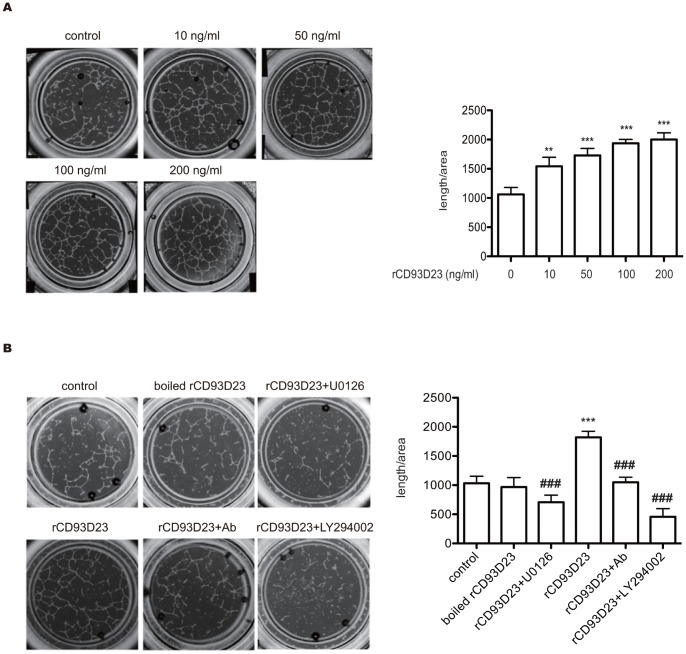
rCD93D23 induces tube formation through the activation of Akt and ERK in HUVECs on Matrigel *in vitro*. (A) rCD93D23 induced tube formation in a dose-dependent manner. (B) The tube formation induced by boiled rCD93D23, and rCD93D23 in the presence of polyclonal anti-CD93D23 IgG (10 µg/ml), U0126 (10 µM), or LY294002 (10 µM). Each value represents the mean ± SD (n = 3), and similar results were obtained in three independent experiments. **, *p*<0.01; ***, *p*<0.001 vs. control. ###, *p*<0.001 vs. rCD93D23.

The role of CD93 in angiogenesis was assessed by the morphological differentiation of HUVECs on Matrigel. Capillary-like structures formed complete networks after 6 h incubation in the presence of different concentrations of rCD93D23 (Fig. 4A), whereas only a small number of tubes were formed in the PBS control. As Fig. 4A indicates, rCD93D23 markedly induced a dose-dependent response of tube formation in HUVECs. The stimulatory effect of rCD93D23 (50 ng/ml) on tube formation in HUVECs was blocked by polyclonal anti-CD93 IgG (10 µg/ml), 10 µM U0126, and 10 µM LY294002 (Fig. 4B). The stimulatory effect of tube formation induced by rCD93D23 disappeared when the rCD93D23 was boiled (Fig. 4B). These results indicated that rCD93D23 had a novel angiogenic activity mediated through the ERK1/2 and PI3K/Akt/eNOS pathways.

### rCD93D23 Promotes Angiogenesis *in vivo*

**Figure 5:**
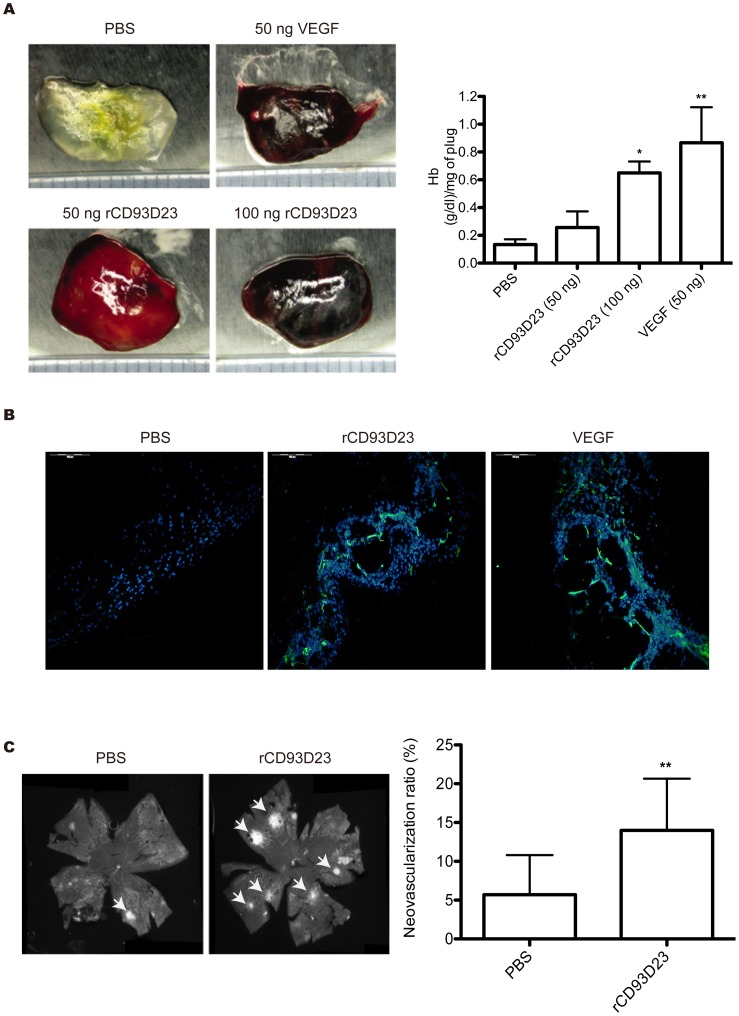
rCD93D23 stimulates angiogenesis *in vivo*. (A) FVB mice were each injected subcutaneously with Matrigel containing VEGF or rCD93D23 near the abdominal midline. The Matrigel plugs were excised after 4 days and then photographed. The Hb content of the excised Matrigel plugs was examined. Each value represents the mean ± SD (n = 3), and similar results were obtained in three independent experiments. *, *p*<0.05 vs. PBS; **, *p*<0.01 vs. PBS. (B) Immunofluorescent staining of Matrigel plugs. Green channel, CD31 staining indicated vessel architecture. Blue channel, DAPI indicated nucleus. VEGF was used as a positive control. (C) The hyperoxia-induced retina vessel loss mice were intraperitoneally injected with rCD93D23 (160 µg/kg) (retina numbers = 20) or PBS (retina numbers = 7) twice a day for 2 days. Subsequently, the mice were sacrificed to harvest the retinas for isolectin staining. The vessel density was quantitated by Photoshop software and analyzed by two-tailed Student’s *t* test. The results were pooled from three independent experiments, all of which had similar results. **, *p*<0.01 vs. PBS. The white arrows indicated the neovascularization tufts.

To further investigate the angiogenic activity of rCD93D23 *in vivo*, the effect of rCD93D23 on blood vessel formation was examined by a murine angiogenesis assay and an oxygen-induced retinopathy (OIR) assay. Matrigel can serve as a vehicle for the slow release of angiogenic factors [19], [20]. The results showed that angiogenesis significantly increased in Matrigel plugs containing 100 ng rCD93D23 or 50 ng vascular endothelial growth factor (VEGF) (Fig. 5A). The degree of angiogenesis was also determined by measuring the hemoglobin (Hb) content of the recovered Matrigel plugs. As shown in Fig. 5A, the Hb content of the Matrigel containing rCD93D23 or VEGF plus heparin had significantly increased compared to that of the control. Moreover, blood vessels were apparently observed in the plugs containing rCD93D23 or VEGF by CD31 immunofluorescent staining (Fig. 5B). OIR is a well-established model to investigate the temporal expression pattern of angiogenesis in hyperoxia induced retinal vessel loss in the neonatal mice [21]. The results showed that administration of rCD93D23 into the hyperoxia treated mice could significantly induce faster retinal vessel regrowth and neovascularization than the PBS control (Fig. 5C). These results suggested that rCD93D23 could promote angiogenesis *in vivo*.

### The EGF-like Domain is Essential for CD93 to Induce Angiogenesis

We further tested whether rCD93D123 also has angiogenic effect in the plug. rCD93D123-containing Matrigels were subcutaneously injected into mice and the plugs at different time points were harvested (days 1, 3, and 5). We extracted total proteins in the plugs and performed a SDS-PAGE to analyze the expression pattern of rCD93D123 by using c-Myc monoclonal Ab, which recognized the c-Myc tag at the C-terminus of rCD93D123. In comparison with the rCD93D123 protein control, the injected rCD93D123 in the Matrigel was processed at day 1 after being injected into mice and the amount of recovered protein decreased at day 3 and day 5 (Fig. S2A), while the Matrigel containing no rCD93D123 protein revealed no positive band following the same extraction procedure. These data indicate that rCD93D123 may be processed into different length of fragments. Furthermore, a murine angiogenesis assay and an OIR assay were performed to test whether the EGF-like domain is essential for CD93 to induce angiogenesis *in vivo*. Both rCD93D23 and rCD93D123 could significantly induce angiogenesis in Matrigel and neovascularization in hyperoxia-treated retina and rCD93D23 had a higher potency than rCD93D123. However, rCD93D1 could not induce angiogenesis *in vivo* (Fig. S2, B and C). These data suggest that the EGF-like domain is essential for CD93 to induce angiogenesis.

### rCD93D23 Activates EGF Receptor (EGFR)

Because rCD93D23 is an EGF-like domain-containing recombinant protein, we tested whether rCD93D23 can bind to EGFR, which is the key signaling transduction pathway in angiogenesis, and examined the downstream ERK phosphorylation. Fig. S3 showed that rCD93D23 or rCD93D123, but not rCD93D1, could bind to EGFR by a co-immunoprecipitation assay (Fig. S3A). Furthermore, blockage of EGFR activity by 10 µM of AG1478 could significantly inhibit rCD93D23- or rCD93D123-induced ERK phosphorylation (Fig. S3B). These data indicate that EGFR could be one of the potential receptors on endothelial cells for rCD93D23-mediated angiogenesis.

## Discussion

CD93 knockout mice have no abnormalities in vascular development [22], suggesting that CD93 is not essential in vasculogenesis and embryonic development. However, CD93 is dominantly expressed in the endothelium [23], and soluble CD93 fragments were detected in the circulation of patients with cardiovascular disease [18]. The roles of soluble CD93 in endothelium are not investigated. Our data first demonstrated, for the first time, that rCD93D23 with the EGF-like domain is a novel angiogenic factor. rCD93D23 can stimulate proliferation, migration, and *in vitro* tube formation in HUVECs (Fig. 1, 2, and 4). It also activates PI3K/Akt/eNOS and EKR1/2 (Fig. 3) signaling pathways that are involved in a number of cellular functions, including cell survival, migration, and angiogenesis [3], [24]. Moreover, rCD93D23 can significantly induce angiogenesis *in vivo* (Fig. 5). Our findings should encourage further investigations into the roles of CD93 in angiogenesis-related diseases.

Previous studies showed that CD93 might contribute to macrophage-mediated phagocytosis of apoptotic cells *in vivo* [22]. The CD93 ectodomain, with an N-terminal lectin-like domain and EGF-like repeats, is susceptible to the MMP-mediated shedding from the surface of human monocytes and neutrophils. Shedding is induced by PKC activators such as phorbol dibutyrate and phorbol myristate acetate [25], [26]. These observations indicate that soluble CD93 may participate in inflammation, especially in the removal of damaged cells. Furthermore, various forms of CD93 ectodomains were detected in the plasma of healthy human donors, and incubation with proinflammatory cytokines such as TNF-α or the lipopolysaccharide induced CD93 shedding from monocytes [25]. These findings suggest that monocytes may be the source of soluble CD93s and that membrane-bound CD93 may be shed in response to inflammation-associated mediators.

Shedding mechanisms have been described for several transmembrane proteins that contribute to the process of angiogenesis. Membrane-anchored forms of TGF-α, amphiregulin, and heparin-binding EGF, which belongs to a prototypical form of the EGF family, act as autocrine growth factors by activating the EGF receptor [27]. Adhesion molecules like E-selectin and vascular cell adhesion molecule-1 can promote angiogenesis in their soluble forms [28]. Because CD93 fragments can be detected in human plasma, it is likely that CD93 is also cleaved under physiological conditions. Although the exact structure of soluble CD93 remains unknown, soluble CD93s seem to include EGF-like domains (D2), as observed from the results of sandwich ELISAs with monoclonal Abs R139 and R3 [5]. To this notion, we subcutaneously injected rCD93D123-containing Matrigel into mice to check whether rCD93D123 could be processed during angiogenesis. We found that the injected rCD93D123 was processed at day 1 after being injected into mice (Fig. S2A). However, in this assay we could only detect c-Myc tagged fragments of CD93. These data indicate that rCD93D123 may be processed into different length of fragments, which may contain the EGF-like domain in the plugs. Furthermore, these EGF-like domain-containing fragments can exhibit angiogenic ability to locally trigger angiogenesis. These observations are consistent with previous reports that the concentration of EGF-like domain-containing CD93 fragments elevated in the plasma of patients with coronary artery disease [18]. It has been reported that CD93 shedding was mediated by an MMP, but was independent of the TNF-α-converting enzyme [25]. Whether MMP-mediated cleavage of CD93 functions in angiogenesis through a paracrine or an autocrine mechanism remains to be studied. Furthermore, whether these natural soluble CD93 subspecies mimic the mitogenic and/or angiogenic properties observed for rCD93D23 is unknown. Our results demonstrate that the EGF-like domain is essential for soluble CD93s to exhibit pro-angiogenic effects on the endothelium. However, the possibility that membrane-intercalated, intact CD93 exerts other biological effects cannot be excluded.

EGFR plays an important role during angiogenesis, and it can sense angiogenic factors stimulation to elicit downstream signaling molecules activation [29]. Increasing reports showed that some EGF-like domains acted as EGFR agonists [30]. In our observations, we showed that rCD93D23 could induce FAK, ERK, Akt, and eNOS phosphorylation in a dose-dependent and time-dependent manner (Fig. 3, A and B). Since CD93 is also a multiple EGF-like repeats containing glycoprotein, it makes us speculate whether CD93 can induce angiogenesis through EGFR activation. Consistent with this notion, we found that rCD93D23 or rCD93D123, but not rCD93D1, could bind to EGFR (Fig. S3A) and activate downstream ERK phosphorylation (Fig. S3B). In addition, our present data showed that rCD93D23 had higher angiogenic activity than rCD93D123, including promotion of cell proliferation and migration (Fig. 1 and 2) and stimulation of angiogenic associated signaling (Fig. 3) *in vitro*. Furthermore, a murine angiogenesis assay and an OIR assay were performed to show that rCD93D1 could not induce angiogenesis *in vivo* (Fig. S2, B and C). It implies that the CTLD of CD93 may have an attenuating role on CD93-mediated angiogenesis. These observations are consistent with our recent publication that CTLD of recombinant thrombomodulin plays a suppressing role on angiogenesis [31].

We demonstrated that soluble CD93s with an EGF-like domain possessed angiogenesis-promoting activity (Fig. 5). Elevated soluble CD93 may be a biomarker for coronary artery disease [18]. We found that rCD93D23 could significantly induce angiogenesis in a murine Matrigel-plug assay (Fig. 5, A and B), suggesting that soluble CD93s with an EGF-like domain have an important role in eliciting angiogenesis. As shown in Fig. 5C, we observed that the rCD93D23 not only promoted vessel re-growth but also induced immature vessel tufts. Generally, the vessels generated by neovascularization in retinopathy animal models are more leaky and immature than the regrowth vessels [32]. However, we have not confirmed the role of soluble CD93s in physiological or pathological neovascularization.

Angiogenesis contributes to metastasis [3] and the development of inflammatory diseases such as rheumatoid arthritis [33], [34]. Therefore, the therapeutic correction of angiogenesis, by preventing disordered angiogenesis, is a potentially fruitful approach for the treatment of a number of human diseases. Our study is the first to report that CD93 domains can promote angiogenesis and that they do so predominantly through the PI3K/Akt/eNOS and ERK1/2 pathways. We suggest that further assessment of the involvement of CD93 in pathophysiological angiogenesis be conducted, and further studies on the potential of rCD93D23 as a novel therapeutic agent for ischemia-related diseases are warranted.

## Materials and Methods

### Expression and Purification of rCD93 Domain Proteins

HUVECs cDNA was used as template in a PCR; a fragment coding for rCD93D1 (residues Thr^22^ through Gly^177^) was amplified by using D1 forward (5′_CCCAAGCTTGGGATGGCCACCTCCATGGGCCTGCTGC_3′) and D1 reverse (5′_GCTCTAGAGCGCCTTTGAAGCTGAACTTGCACACG_3′) primers; a fragment coding for CD93D23 (residues Val^258^ through Lys^580^) was amplified by using D23 forward (5′_TTGGAATTCCCCAAGTATGGCTGCAACTTC_3′) and D3 reverse (5′_AATTCTAGATACTTTTGCCCGTCAGTGCCA_3′) primers. The rCD93D123 fragment (residues Thr^22^ through Lys^580^) was amplified by using rCD93D123 forward (5′_CCCAAGCTTGGGATGGCCACCTCCATGGGCCTGCTGC_3′) and D3 reverse primers. The amplified DNA fragments were constructed into the pCR3 vector (Invitrogen) with c-Myc as a tag for protein detection to generate recombinant human rCD93D1, rCD93D23, and rCD93D123 in the mammalian HEK293 expression system. Conditioned medium containing secreted rCD93D1, rCD93D23, or rCD93D123 was applied to a nickel-chelating Sepharose column (Amersham Pharmacia Biotech), and rCD93D1-, rCD93D23-, or rCD93D123-containing fractions were collected by gradient elution with 300 mM imidazole (Sigma-Aldrich). The endotoxin of recombinant proteins were assessed by the Limulus Amoebocyte Lysates assay, and the endotoxin levels were lower than 1.0 EU/µg.

### Characterization of rCD93 Domain Proteins

rCD93D1, rCD93D23, and rCD93D123 were verified by LC-MS/MS and the data was searched using MASCOT software (Matrixscience) against the NCBI database (Fig. S1A). The LC-MS/MS data of rCD93D23 and rCD93D123 were generated by using an electrospray ionization-Trap mass spectrometer (ThermoFinnigan; LCQ DECA XP Plus) which was equipped with a LC system (Perkin Elmer; Series 200). A C_18_ LC column (150×0.075 mm, 3.5 µm; Agilent, ZORBAX 300SB-C18) was used. The extracted peptides were dissolved in an A buffer (H_2_O with 0.01% formic acid). A fifty-minutes gradient was used, ramping from 0% to 100% B buffer (100% acetonitrile with 0.01% formic acid) in three-linear gradient steps to elute peptides. The raw data files were submitted to MASCOT (version 2.1; Matrix Science) for protein identification. The error tolerance of peptide mass and fragment mass was set to ±1Da. The potential glycosylation sites were predicted by NetOGlyC 3.1 software (Fig. S1B). The sequencing informations of the overexpression vectors (pCR3 vectors with domains 2 and 3, or domains 1, 2, and 3 of CD93) also have been presented by Vector NTI software (Invitrogen) (Fig. S1C).

### Cell Culture

HUVECs were purchased from Life Technologies Corporation. The cells obtained through source facilities are consistent with the applicable legal and ethical practices of the United States. The cells from the second or third passages were used in all experiments.

### Cell Proliferation Assay

To assess the ability of rCD93 domain proteins to stimulate HUVECs proliferation, tetrazolium salt WST-1 (4-[3-(4-iodophenyl)-2-(4-nitrophenyl)-2H-5-tetrazolio]-1, 3-benzene disulfonate) was used in accordance with the manufacturer’s instruction (Roche). Briefly, HUVECs were treated with various concentrations of rCD93D1, rCD93D23, rCD93D123, or VEGF (10 ng/ml) (R&D Systems) for 48 h. WST-1 was added to each well, and the absorbance was measured at a wavelength of 450 nm after 1.5 h of incubation. In addition, the actual cell numbers were also manually counted to accurately show the effects of recombinant proteins on cell proliferation.

### Cell Migration Assay

HUVECs migration was measured using Transwell (Corning Costar Corp.) with 6.5-mm-diameter polycarbonate filters (8-µM pore size). The lower surface of each filter was coated with 500 µg/ml gelatin (Sigma-Aldrich). Different concentrations of rCD93 domain proteins or 10 ng/ml VEGF were diluted in M199 containing 1% FBS, and the mixtures were placed in the lower wells. HUVECs were seeded into each upper well at a density of 1×10^5^ in 100 µl of M199 medium containing 1% FBS and were allowed to migrate for 4 h at 37°C. The cells were fixed with methanol and stained with Giemsa (Sigma-Aldrich). The total migrated cells were counted with an optical microscope (Olympus). For the rCD93D23 functional test, rCD93D23 in the absence or presence of 5 µg/ml mouse polyclonal anti-CD93D23 Ab, which was prepared in our laboratory from BALB/c mice immunized with rCD93D23 protein was used to perform the migration assay. To verify the role of ERK1/2 and PI3K/Akt in rCD93D23-mediated cell migration, assays were performed in the presence of U0126 (Promega), an MEK inhibitor, and LY294002 (Calbiochem), a PI3K inhibitor.

### Assay of ERK1/2, Akt, eNOS, and FAK Phosphorylation

HUVECs were cultured to confluence in a 6-cm-diameter dish and were incubated in M199 containing 1% FBS for 18 h. After washing with PBS, cells were incubated with serum-free M199 for 6 h and were treated with the indicated concentrations of rCD93D23. Cell lysates were separated by SDS-PAGE, and the levels of phospho-ERK1/2 (Tyr-204) and total ERK1/2, phospho-Akt (Ser-473) and total Akt, phospho-eNOS (Ser-1177) and total eNOS, and phospho-FAK (Tyr-397) and total FAK were analyzed by western blotting with specific antibodies (Cell Signaling Technology). To verify the role of EGFR in rCD93D23-mediated ERK activation, assay was performed in the presence of AG1478 (Millipore), an EGFR inhibitor.

### Zymography Assay

HUVECs were seeded on 10-cm-diameter dishes in supplemented M199 medium. After 24 h, cells were rinsed twice with serum-free M199 and were incubated in serum-free M199 with various concentrations of rCD93D23 or VEGF (10 ng/ml) for 20 h. The conditioned medium containing 5 µg of secreted proteins was analyzed by gelatin-based zymography [35]. The digested area appeared clear on a blue background, indicating the location of MMPs activity.

### *In vitro* Matrigel Angiogenesis Assay

After 16 h of serum and growth factor depletion, HUVECs were resuspended at a density of 1.0×10^5^/ml in M199 containing 4% FBS. Cell suspensions (50 µl) treated with various concentrations of rCD93D23, inhibitors, or VEGF were added to wells of μ-slide Angiogenesis (Integrated BioDiagnostics), which contained 10 µl of growth factor-reduced Matrigel (BD Biosciences). Each concentration was tested in quadruplicate in the same plate, and capillary-like structures were photographed with Olympus camera (×40 magnification).

### Murine Angiogenesis Assay

To analyze the angiogenic effect *in vivo* [19], growth factor-reduced liquid Matrigel (0.5 ml) was mixed with heparin (30 U/ml) and either rCD93D1 (100 ng per plug), rCD93D23 (50 or 100 ng per plug), rCD93D123 (100 ng per plug), VEGF (50 ng per plug), or PBS. The mixture was then subcutaneously injected in FVB mice near the abdominal midline. Four days after injection, mice were euthanized, and Matrigel plugs were surgically removed. For macroscopic analysis of angiogenesis, Hb content in Matrigel was measured with Drabkin reagent 525 (Sigma-Aldrich). For histological analysis, CD31 Ab (Abcam) was used to detect blood vessels.

### OIR Model

The 7-day-old (P7) C57BL/6 mice with nursing mothers were subjected to hyperoxia (75% oxygen) for 5 days, which inhibited retinal vessel growth and led to significant vessel loss. On P12, the mice were returned to room air and intraperitoneally administrated with rCD93D1, rCD93D23, rCD93D123 (160 µg/kg), or PBS twice a day for 2 days. At P14, the mice were sacrificed to harvest the retinas for further analysis. Both the re-growth vessels and retinal neovascularization were stained with isolectin (Invitrogen). The vessel density was quantitated by Photoshop software as described previously [21].

### Co-immunoprecipitation Assay

Subconfluent HUVECs were starved in serum-free M199 for 4 hours, and then incubated with 0.5 **µ**g of rCD93D1 (lane 1), rCD93D23 (lane 2), or rCD93D123 (lane 3) for 30 minutes. The associations of EGFR and recombinant proteins were tested by immunoprecipitating EGFR with EGFR Ab (Santa Cruz Biotechnology) and detecting recombinant proteins by immunoblotting recombinant proteins with c-Myc Ab (Santa Cruz Biotechnology). Rabbit IgG was used as a negative control (lane 4).

### Animal Care

Animal care conditions and design of experiments were approved by the Institutional Animal Care and Use Committee of the National Cheng Kung University (Tainan, Taiwan).

### Statistical Analysis

Data are shown as mean ± SD. Statistical significance was analyzed by one-way ANOVA followed by Bonferroni post hoc test. Two-tailed Student’s *t* test was applied to the OIR model (GraphPad Prism version 5.0. GraphPad software). Differences with *p*-value <0.05 were considered significant.

## Supporting Information

Figure S1 **Bioinformatics searches of rCD93D23 and rCD93D123.** (A) rCD93D23 and rCD93D123 after LC-MS/MS analysis were identified by MASCOT software. (B) The potential *O*-glycosylation sites of CD93 were predicted by NetOGlyC 3.1 software. (C) The sequencing informations of rCD93D23 and rCD93D123 in pCR3 vectors were analyzed by Vector NTI software. Yellow box, signaling peptide. Blue box, start codon. Red box, stop codon.
(TIF)

Figure S2 **The EGF-like domain is essential for CD93 to induce angiogenesis.** (A) The rCD93D123 (0.5 µg) -containing Matrigel plugs were harvested at days 1, 3, and 5. The expression pattern of rCD93D123 in plugs was identified by c-Myc monoclonal Ab. M1, Matrigel only was used as a negative control at day 1. D123, 100 ng of rCD93D123 was used to identify the molecular weight of recombinant protein. (B) FVB mice were each injected subcutaneously with Matrigel containing VEGF, rCD93D1, rCD93D23, or rCD93D123 near the abdominal midline. The Matrigel plugs were excised after 4 days and then photographed. The Hb content of the excised Matrigel plugs was examined. Each value represents the mean ± SD (n = 3), and similar results were obtained in three independent experiments. *, *p*<0.05 vs. PBS; ***, *p*<0.001 vs. PBS. (C) The hyperoxia-induced retina vessel loss mice were intraperitoneally injected with rCD93D1, rCD93D23, or rCD93D123 (160 µg/kg) (retina numbers = 4) or PBS (retina numbers = 3) twice a day for 2 days. Subsequently, the mice were sacrificed to harvest the retinas for isolectin staining. The vessel density was quantitated by Photoshop software. *, *p*<0.05; **, *p*<0.01 vs. PBS. The white arrows indicated the neovascularization tufts.
(TIF)

Figure S3 **rCD93D23 activates EGFR.** (A) rCD93D1, rCD93D23, or rCD93D123 incubated HUVECs lysate were immunoprecipitated with specific EGFR Ab, and then immunoblotted by c-Myc Ab. (B) AG1478 effectively blocked rCD93D23- or rCD93D123-induced ERK phosphorylation. rhEGF, recombinant human EGF, was used as a positive control.
(EPS)
