# Supplementary figures and images for: The Epidermal Growth Factor-like Domain of CD93 Is a Potent Angiogenic Factor
Source: PLoS One. 2012 Dec 18;7(12):e51647. doi: 10.1371/journal.pone.0051647 (PMC3525571; doi:10.1371/journal.pone.0051647)

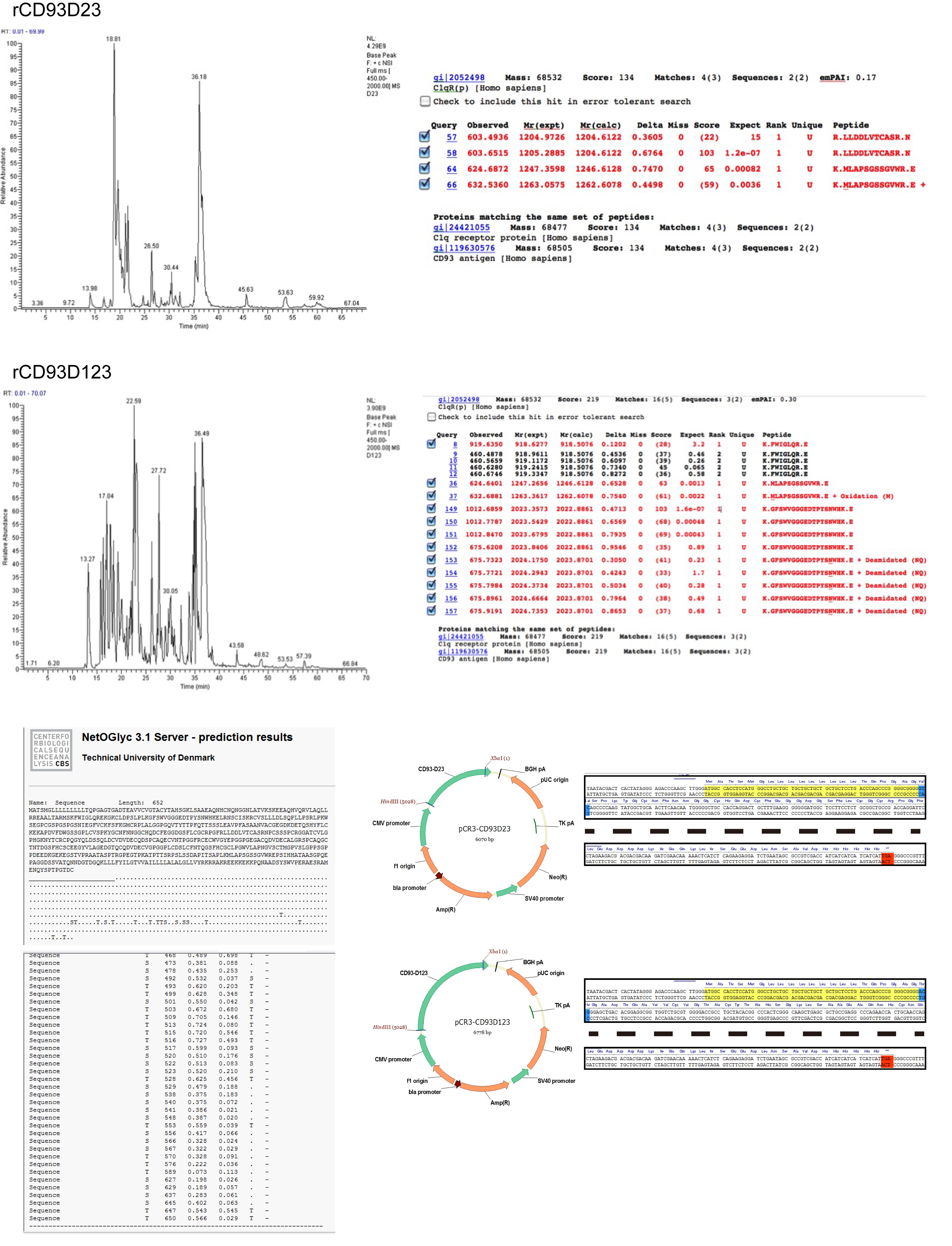

Supplement: Figure S1 — Bioinformatics searches of rCD93D23 and rCD93D123. (A) rCD93D23 and rCD93D123 after LC-MS/MS analysis were identified by MASCOT software. (B) The potential O-glycosylation sites of CD93 were predicted by NetOGlyC 3.1 software. (C) The sequencing informations of rCD93D23 and rCD93D123 in pCR3 vectors were analyzed by Vector NTI software. Yellow box, signaling peptide. Blue box, start codon. Red box, stop codon. (TIF) [file pone.0051647.s001.tif]

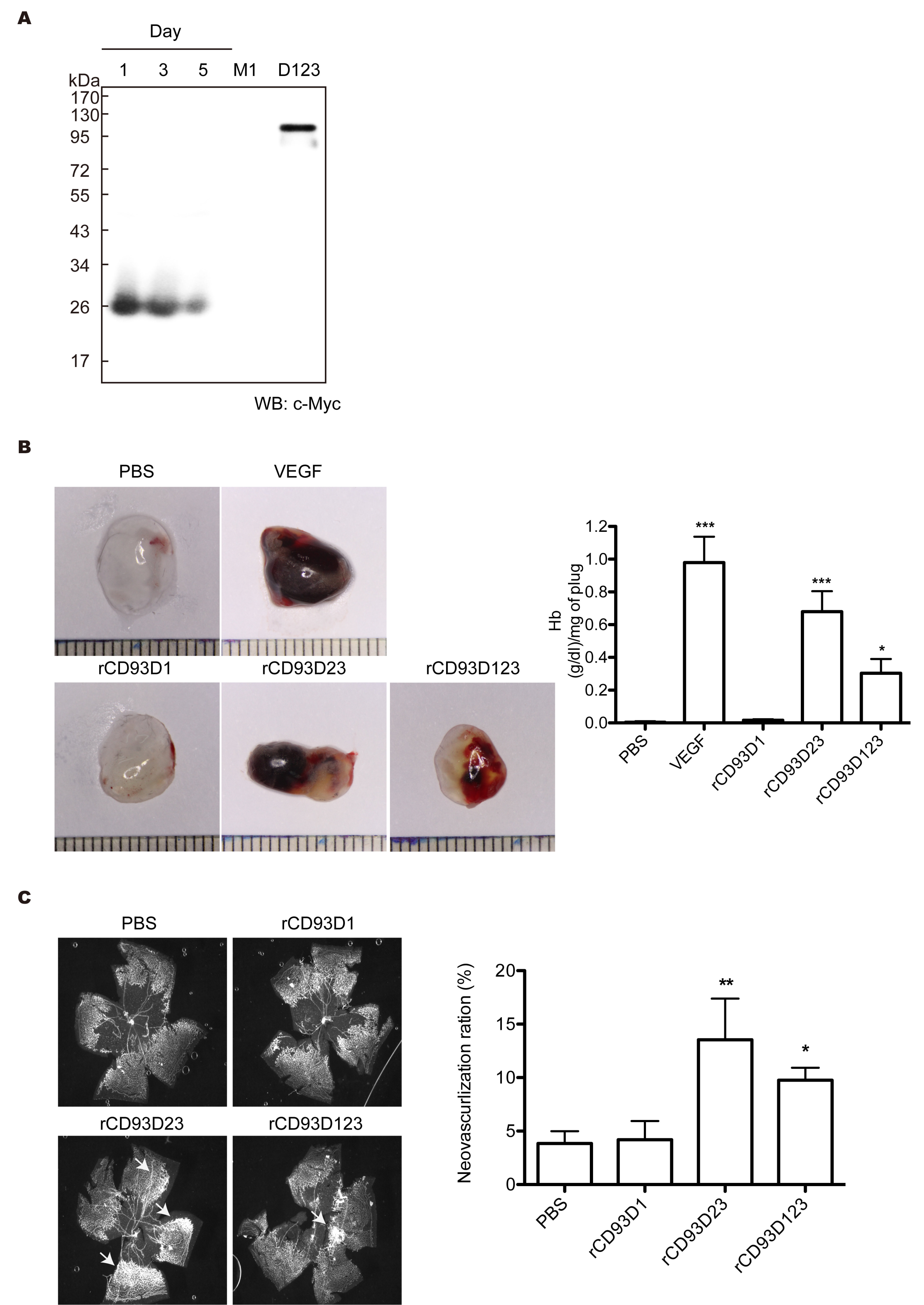

Supplement: Figure S2 — The EGF-like domain is essential for CD93 to induce angiogenesis. (A) The rCD93D123 (0.5 µg) -containing Matrigel plugs were harvested at days 1, 3, and 5. The expression pattern of rCD93D123 in plugs was identified by c-Myc monoclonal Ab. M1, Matrigel only was used as a negative control at day 1. D123, 100 ng of rCD93D123 was used to identify the molecular weight of recombinant protein. (B) FVB mice were each injected subcutaneously with Matrigel containing VEGF, rCD93D1, rCD93D23, or rCD93D123 near the abdominal midline. The Matrigel plugs were excised after 4 days and then photographed. The Hb content of the excised Matrigel plugs was examined. Each value represents the mean ± SD (n = 3), and similar results were obtained in three independent experiments. *, p<0.05 vs. PBS; ***, p<0.001 vs. PBS. (C) The hyperoxia-induced retina vessel loss mice were intraperitoneally injected with rCD93D1, rCD93D23, or rCD93D123 (160 µg/kg) (retina numbers = 4) or PBS (retina numbers = 3) twice a day for 2 days. Subsequently, the mice were sacrificed to harvest the retinas for isolectin staining. The vessel density was quantitated by Photoshop software. *, p<0.05; **, p<0.01 vs. PBS. The white arrows indicated the neovascularization tufts. (TIF) [file pone.0051647.s002.tif]
